# Supplementary material for: MVP: a modular viromics pipeline to identify, filter, cluster, annotate, and bin viruses from metagenomes
Source: mSystems. 2024 Oct 1;9(10):e00888-24. doi: 10.1128/msystems.00888-24 (PMC11498083; doi:10.1128/msystems.00888-24)
Supplement: Supplemental legends — Legends for supplemental figures and tables. [file msystems.00888-24-s0002.docx]

**SUPPLEMENTARY LEGENDS**

**Supplementary Figure S1. Sampling sites and collection methods from the Loxahatchee Nature Preserve.** Map of locations of sampling sites (S1A). Sampling collection and environmental conditions (S1B). Credits: <https://jgi.doe.gov/inspiring-stem-careers-hands-on-everglades-loxahatchee-microbiome/> (Alexander Klimczak).

**Supplementary Figure S2. Quality assessment of viral contigs (S2A) and vOTUs (S2B) using CheckV.** The x-axis represents the length of viral contigs or vOTUs in kilobase pairs, while the y-axis indicates the CheckV quality categories: not-determined, low-quality, medium-quality, high-quality, and complete.

**Supplementary Figure S3. Non-metric multidimensional scaling (nMDS) ordination plots showing beta-diversity of viral communities.** The nMDS plot illustrates the differences in viral community composition among the four locations, with R² and p-values indicating the significance of the differences observed, for unfiltered horizontal coverage (HC) (S3A), 50% filtered HC (S3B), 90 % filtered HC (S3C) vOTU communities, unfiltered horizontal coverage (HC) vBins and unbinned vOTUs (S3D), unfiltered horizontal coverage (HC) vBins (S3E), 10% horizontal coverage (HC) vBins and unbinned vOTUs (S3F), 50% horizontal coverage (HC) vBins and unbinned vOTUs (S3G), 90% horizontal coverage (HC) vBins and unbinned vOTUs (S3H.)

**Supplementary Figure S4. Overview of viromics analysis using ViWrap. S4A.** Number of viral contigs identified under different filtration criteria (Conservative, Relaxed, and Unfiltered) across various locations (Lox_East, Lox_North, Lox_South, Lox_West). **S4B.** Number of vOTUs identified under conservative, relaxed, and unfiltered conditions. **S4C.** Quality assessment of unbinned vOTUs and vBins using CheckV. **S4D.** Distribution of vOTUs based on taxonomic assignment (Taxonomically assigned vs. Unknown). **S4E.** Host assignment of vOTUs, indicating the number of vOTUs assigned to specific host taxa.

**Supplementary Table 1.** Standard scores and E-values cutoffs used for each database

**Supplementary Table 2.** Sequencing and assembly statistics for 20 samples (4 sites, with 5 replicates each) from the Loxahatchee Nature Preserve

**Supplementary Table 3.** Binning statistics for 20 samples
